# Formal Specification and Design Techniques for Wireless Sensor and Actuator Networks

**DOI:** 10.3390/s110101059

**Published:** 2011-01-19

**Authors:** Diego Martínez, Apolinar González, Francisco Blanes, Raúl Aquino, José Simo, Alfons Crespo

**Affiliations:** 1 Department of Automation and Electronics, Autonomous University of the West, Cll 25 # 115 - 85 Km. 2 Vía Cali-Jamundí, Colombia; 2 Faculty of Mechanical and Electrical Engineering, University of Colima, Av., Universidad # 333, 28000 Colima, Mexico; 3 Department of Computer Engineering (DISCA), Polytechnic University of Valence, Camino de Vera s/n, Valencia, Spain

**Keywords:** sensor networks, wireless control networks, Colored Petri Nets

## Abstract

A current trend in the development and implementation of industrial applications is to use wireless networks to communicate the system nodes, mainly to increase application flexibility, reliability and portability, as well as to reduce the implementation cost. However, the nondeterministic and concurrent behavior of distributed systems makes their analysis and design complex, often resulting in less than satisfactory performance in simulation and test bed scenarios, which is caused by using imprecise models to analyze, validate and design these systems. Moreover, there are some simulation platforms that do not support these models. This paper presents a design and validation method for Wireless Sensor and Actuator Networks (WSAN) which is supported on a minimal set of wireless components represented in Colored Petri Nets (CPN). In summary, the model presented allows users to verify the design properties and structural behavior of the system.

## 1. Introduction

Wireless sensor and actuator networks are used to improve control system efficiency through several functions that are distributed over different wireless nodes. Control systems wherein the control loops are closed through networks are called Networked Control Systems (NCS). The NCS implementation helps reduce failure impacts in the system components and facilitates process analysis, maintenance and traceability. The use of wireless networks to communicate the system nodes enables researchers to develop new applications on wireless sensor and actuator networks (WSAN) that increase flexibility, reliability and portability, while at the same time significantly decreasing their cost. The MAC (Medium Access Control) mechanism used by the network determines the delay and jitter that occur during the transmission period, which produce sometimes discrepancy between experimental and simulations results. This is because the models used to analyze and design these systems often use inadequate validation methods. Moreover, there are some technologies that do not support the models correctly.

**Figure 1.**
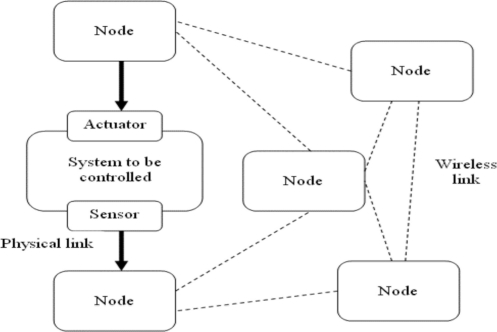
System general structure.

In these applications, the sensor, controller and actuator functions are distributed at the system nodes. As a result of physical connections, some functions have pre-assigned locations, which is the case of sensors and actuators. Meanwhile, other functions can be assigned to other nodes according to specified criteria such as time constraints, power consumption optimization and delays reduction, among others, [1,2]. Figure 1 presents a general structure of these applications.

The nondeterministic and concurrent behavior of distributed systems makes their analysis and design complex. These systems may change states frequently and the specific operating conditions under which they must work are not likely to be considered during design phases. These two considerations sometimes lead to undesirable system performance and are the primary reasons because experimental results and the proposed control objectives sometimes do not correlate. This lack of concordance is often due to imprecise models used to analyze, validate and design systems and platforms that do not support the models used.

In summary, the development of prototypes is difficult and with a high degree of investment in time, requiring experience in software development, hardware and the application domain. It is desirable to use a coherent process to construct these systems, which contain the necessary elements for its realization. This paper presents a method to design and validate WSAN applications using CPN (Colored Petri Nets). The method discussed define a model of computation for wireless sensor and actuator networks from an architectural design based on predefined components, and a specification based on CPN from which is easy to generate the equivalent in C language. The computational model explicitly captures timing information, allows systems to be represented at different levels of granularity, and improves expressiveness by allowing tokens to carry information. It will allow the validation of some properties of the final implementation.

The paper is organized as follows: Section 2 presents a literature review of related work. Section 3 discusses our node architecture and Section 4 presents our design procedure proposed for WSAN. Results of analysis of a case study are presented in Section 5. Finally, Section 6 presents conclusions and future work.

## 2. Related Works

This work integrates several aspects from wireless network protocols, end-to-end real-time analysis, dynamic voltage analysis and design methodologies. In this section, we provide a discussion related literature.

### 2.1. Networks Protocols

Several important articles [3,4], discuss using Bluetooth, IEEE 802.11b and 802.11e technologies to implement control systems. Their results show that IEEE 802.11e EDCA (Enhanced Distributed Channel Access) mode allows to fulfill with real-time constrains in industrial applications for less than 10 nodes and sample periods upper than 10 ms. Other important papers are [5,6].

Pantazis *et al*. [7] examines several methods to reduce the power consumption at different levels of the communication stack in wireless sensor networks. One important method is Time Division Multiple Access (TDMA), which ensures time transmission bounds, minimizes collisions, and facilitates the implementation of energy-saving strategies.

Currently, there are several commercial devices that use Zigbee (Zigbee Specification) [8] and WirelessHART [9], which consume less power than systems supported by IEEE 802.11. Zigbee is supported by the IEEE 802.15.4 standard which provides asynchronous and synchronous (through beacons) media access. The advantage of the asynchronous MAC algorithm is that it facilitates scalability and network auto-configuration; however, it does not fully guarantee time transmission bounds. In the IEEE 802.15.4 synchronization mode, the maximum time to transmit information can be delimited using guaranteed time slots (GTS) within a super frame. It is possible to assign a maximum of seven slots with a minimum frame period of 15.36 ms, which may be enough in some cases. However, the use of GTS is restricted to networks with a star topology, which limits the reliability and scalability of the application.

Control applications in industrial environments require reliable and secure communications. These requirements are easier to provide by networks with a mesh topology, which also benefits the scalability of applications. In order to construct mesh network architecture, Zigbee uses a MAC algorithm without synchronization. Nevertheless, some proposals have been developed to construct synchronized cluster tree networks as presented in [10].

WirelessHART is based on the physical layer of IEEE 802.15.4-2006, but specifies new levels of data link, network, transport and application, [11]. WirelessHART uses a MAC mode in TDMA, with 100 slots per second. Additionally, WirelessHART allows developing mesh topology networks that provide redundant paths, permit routing messages through different routes to avoid broken links, interference, and physical obstacles.

### 2.2. Scheduling Algorithms for Messages and Real Time Tasks

Because delays affect the performance of NCS, their applications have end-to-end real-time constraints. The problem of assessing the feasibility of a real time distributed system is NP-hard. In order to overcome this inherent difficulty, problem restrictions and heuristics must be used. A common approach is to statically allocate application tasks at system nodes and locally utilize either a well known scheduling algorithm like Rate Monotonic (RM) or Earliest Deadline First (EDF), [12].

Applications on distributed systems are characterized by precedence relationship between their tasks. If the tasks are statically allocated to their corresponding processors, end-to-end time constraints can be analyzed by a theory which assumes release jitter [13]. Several studies have been developed to analyze end-to-end scheduling, which uses task scheduling algorithms like RM and EDF, and MAC protocols based on TDMA, Token and Priorities [14–16]. These works use buffers to store messages in network nodes and employ a scheduling method to deliver messages. They are based on finding the maximum response time of all messages.

As far as wireless sensor networks are concerned, one of the most commonly used operating systems is TinyOS [17], which was designed to be used in systems with limited resources, such as 8-bit micro-controllers. TinyOS is supported by a programming model based on components and guided by events, with event handlers having a higher priority than tasks, which are executed based on scheduling policy First-Come First-Served (FCFS). However, such schedulers are not appropriate for real time systems. Zigbee products from Chipcon and Texas Instruments use a scheduler based on static priorities.

Although fixed priority scheduling is the most popular on-line scheduling policy in real-time systems, the EDF policy is gaining greater acceptance in industrial environments because of its benefits in the use of systems resources. EDF is currently available in real-time languages such as RTSJ. It is also available in real-time operating systems like SHark and Erika.

### 2.3. Dynamic Voltage Scaling

Dynamic Voltage Scaling (DVS) is an important strategy used to power-aware in embedded systems like WSN nodes. Several papers have been written about using DVS to meet real-time constraints. [18] presents a methodology based on heuristics for DVS which requires a low computation time and [12] provides a method to achieve optimal operating frequency with minimum power consumption. However, this method is very complex and is not appropriate for online use. [19] proposes a method that combines DVS with a scheduling policy based on a task elastic model which, according to various performance targets, adjusts the period of the system tasks. [20] uses feedback control scheduling for the DVS processor and an EDF scheduler to schedule tasks.

Pillai *et al*. [21] evaluates the performance of several algorithms, including static voltage scaling, which selects the lowest operation frequency to accomplish real time constraints. By using static voltage scaling, the operation frequency is assigned statically and is not modified unless the tasks set change. The advantages of this method are its easy implementation and a very low computational load. However, it is very restrictive because it uses worst case execution time so, consequently, it does not provide the greatest energy savings.

### 2.4. Design Methodologies for Wireless Sensor and Actuator Networks

Meshkova *et al*. [22] proposes a design methodology for wireless sensor networks (WSN) that is service-oriented, but there are no tools to automate the proposed procedure. This methodology is based on the interaction between components to jointly provide a service based on specified criteria. The stages of the design methodology include system requirements, analysis, solution design, and development of software architecture, code development, implementation and testing.

The main parameters considered for wireless sensor networks are cost, lifetime, delay, fault tolerance and services. Additionally, network layer considerations include expected node mobility, failure rate, minimum bandwidth requirements, node number and density, and the average network diameter, as well as network symmetry and heterogeneity. The service parameters includes a list of planned features and facilities, for which each service is characterized by the quality of service expected. Regarding the hardware and software specification, a hardware platform and sensor, the operating system required, the amount of memory needed, and a list of modules required to operate the software are proposed.

Bonivento *et al*. [23,24] present a design procedure for industrial WSN, which begins with a high level description of the control algorithm and a set of possible hardware platforms for the itself synthesis, later to automatically generate an application to fulfill with system requirements and optimize energy consumption. To manage the heterogeneity and complexity, three abstraction layers and tools are introduced to facilitate the transition between different layers and obtain the final solution. This approach to system-level design is characterized by a top-down phase where application requirements are detailed on the end-to-end network constrains, a lower phase which abstracts the hardware performance, and middle phase where the previous two phases can be met. The middle phase utilizes requirements and performance to solve a constrained optimization problem whose solution determines the medium access protocols and routing of the network. Three virtual components are proposed to describe the application, sensors, controllers and actuators; however, the component model restrictions to a system level limit the proposed solution. For example, it does not provide communications between virtual sensors, which is necessary in sensorial fusion applications. Additionally, this procedure separates network and embedded system design, which limits system optimization parameters like power consumption, delays and jitter, among others. The protocol presented for media access is implemented in two different levels. The upper level uses the TDMA forwarding strategy to communicate sensor groups in order to save energy and reduce data collisions. Meanwhile, the lower level uses contention access, where nodes exchange information within each group. This, however, does not ensure maximum delivery of information. In addition, the routing algorithm uses a strategy to discover the shortest path, which is not always the best option in terms of energy savings and communication delays.

Prasad *et al*. [25] presents an analysis and design method for WSN which is supported by the ANDES tool. The method consists of an in-depth analysis prior to system implementation, which is supported by theoretical analysis techniques to estimate the most important parameters of system performance, including network lifetime, coverage and reliability. The method also includes an intuitive analysis of real-time network constraints supported by the network capacity, which relates the distance messages must travel. These theoretical analysis techniques are supported by a set of system parameters that include the number of nodes and the detection range, as well as the available bandwidth. During the design process, these theoretical analysis techniques can be applied iteratively to adjust system parameters in function of the desired performance and the estimated yield. However, the tool does not cover all of the design phases of these applications.

In [26] was presented an application-level design methodology for WSANs in mobile control applications. Which use a simple method to predict from previous control command values on the actuator nodes to cope with packet loss occurring in WSANs. This approach is different from previous because it finds the solution at algorithm level but not offers guaranty of fulfill with time constrains.

## 3. Nodes Architecture

**Figure 2.**
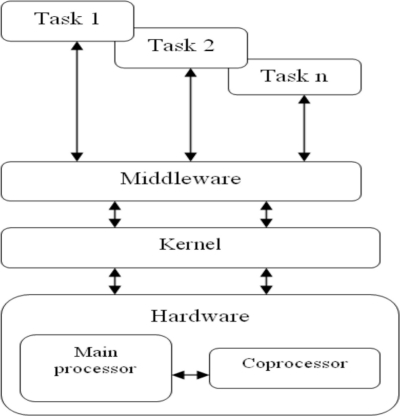
Logical architecture for nodes.

In [1,2] was presented a node architecture for WSN, Figure 2. Design of these systems is characterized by application constraints, this architecture is designed for applications that have a data transmission rate of less than 250 kbps, low computational requirements and operate in small areas. It also meets real time constraints and facilitates the implementation of energy-saving strategies.

The different levels of the architecture have specific roles:

- Tasks: perform activities related to the application.
- Middleware: receives requests from tasks to execute a predefined function, understanding it as a set of tasks with known computational requirements and constraints. The middleware then sends commands to the kernel, which based on a static voltage scaling algorithm, [23], sets the correct values of frequency and voltage to operate the processor and then selects the tasks to be executed by the kernel. In order to activate tasks for each application scenario and considering than in these applications the number of tasks unassigned to a specific node and its code is reduced, it is supposed that each node has a replica of every unassigned task which execute depending on the scenario of the system.
- Kernel: executes tasks based on an EDF scheduling policy.
- Hardware: consists of two processors: the main processor and the coprocessor. The main processor executes the software and uses DVS strategies to save energy; processors with XScale architecture can be used for this [27]. The coprocessor integrates the physical, data link and network communications layers so it do not affect the performance of processing functions, thus ensuring the application quality of service and the synchronization of network nodes. The media access control uses a TDMA algorithm, which is appropriate to fulfill real-time constraints and facilitates the implementation of energy saving strategies.

This proposal makes three assumptions: (1) all nodes are linked to the same network, (2) only one hop is required to transmit a message and (3) the message stored in buffers is discarded. Additionally, because the size of messages in industrial applications is small compared to the amount of data supported by each message in current standard protocols (maximum payload in physical layer PDU of 127 bytes for WirelessHART and ZigBee), we also assume that every message is sent within the space reserved for a node in the TDMA network, thus the maximum network delay is equal to the period needed to repeat the guaranteed time slots in the TDMA.

### 3.1. System Model and Notation

By assume a general framework wherein the NCS’s tasks, sensor, controller and actuator, are executed in different nodes, in that sequence and using mutual exclusion, the following functions and concepts are defined

- *T_SF_* is the period needed to repeat guaranteed slots in the TDMA based network.
- *D_CGR_* is the end-to-end deadline measured from sensor task start until actuator task finalize, according to the control performance goals.
- *T_S_* is the sampling period used by the sensor task, which is defined according to the dynamic system and comply with *D_CGR_* ≤ *T_S_*
- τ= {*Task*_1_, *Task*_2_, ..., *Task_n_*}, is a feasible EDF task set with *Task_i_* = (*W CET_i_*, *D_i_*, *P_i_*); *W CET_i_*, *D_i_* and *P_i_* are the respective values of worst case execution time, deadline and period of task *Task_i_*.
- *W CRT_i_* is the worst case response time for a task *Task_i_*.

Martínez *et al*. [1] presents a scheduled test for WSAN multi-hop based on previous nodes architecture and system notation.

## 4. Design of a WSAN

The design of a WSAN consists of two main phases. In the first phase, the components are used to develop the system architecture and in the second phase the verification is performed.

### 4.1. Components

A minimum set of components for the design includes 2 types of activities:

- Periodic, cyclical activities with constant time intervals.
- Aperiodic, activities initiated by the occurrence of external events

Implementation elements. These elements are necessary to establish the interaction between activities:

- EDF scheduler: Represents the behavior of an EDF scheduler and allows to analyze the incidence of expropriation on the task’s computation time.
- TDMA: Represents a network with medium access control TDMA. Allows analyzing delays on sending messages.

### 4.2. Design Validation

A method to verify the system operation is to develop an executable model of it. Through model simulation it is possible to analyze how the system will perform. Additionally, the development of this type of models allows obtaining the complete specifications that provide systematic information about different scenarios, thus greatly reducing the number of design errors.

Petri Nets are graphical and mathematical tools designed to describe and study systems that are concurrent, asynchronous, distributed, parallel, nondeterministic and/or stochastic. Petri Nets makes it possible to describe sequences, conflicts and concurrences. Petri Nets can represent systems with different levels of complexity, which makes it possible to describe and formally validate systems. Graphically, Petri Nets provide an observable manner to determine whether a system will present conflicts during operation. These models can be used for testing during the design phase due to that in final prototype that would be inappropriate, either by cost, time, location in the plant, *etc*. Another important aspect of Petri Nets focuses on modeling the different subsystems of a larger system in the same language, thus facilitating the interaction of different experts who must interact to develop complex applications, such as the WSAN. This is of great importance because of the current state of analysis and development tools. There are tools that do not always integrate the different architecture levels (CPU, OS, Communications Network, Middleware, coprocessors and application features). This lack of integration leads to ad hoc development of the different subsystems that comprise the architecture, making it difficult to obtain an adequate overview of the system during the design phases. Furthermore, although some simulation environments permit the integration of different system architecture levels, these applications do not always model the different components in a manner that is consistent with the desired system characteristics. Petri Nets allow to verify the following properties:

- Behavioral (those that depend on the initial marking): reachability, boundedness, safeness and liveness, reversibility, cover-ability, persistence, fairness.
- Structural (which depend only on the network topology and are independent of the initial marking): liveness structural controllability, structural boundedness, repeatability and consistency.

Among the analysis techniques include:

- Method of tree coverage (reachability): This can be applied to all types of networks, but is usually limited to smaller networks due to the state space explosion.
- Incidence Matrix: This parameter is well suited to analyzing structural properties.
- Reduction or decomposition techniques: Because of their generality, models based on Petri Nets become too large to analyze, even for simple systems. Therefore, it is often necessary to add amendments or restrictions when they are used in any particular application. To facilitate the analysis of large systems can reduce the system model to a simpler one while preserving the properties to analyze. These techniques can be used to transform an abstract model in a more refined in a hierarchical fashion.

Colored Petri Nets (Colored Petri Nets, CP-nets or CPN) have marks whose value represents a specific type of data [28,29]. These formal models can be used to verify the properties of the system, which are supported by a set of state-space methods, from which it is possible to verify the locks absence and the possibility of the system evolving to a particular state. These tests can also be applied to time CP-nets. In addition to the data, it is possible to add a field to represent the instant of time, enabling the verification of temporal aspects of the system [30]. The main disadvantage of analysis methods supported in state-space is the state explosion problem. To address this problem, researchers have generated tools such as CPN Tools [30] that include reduction techniques that bound the problem of states explosion.

### 4.3. Components Representation in CPN

**Figure 3.**
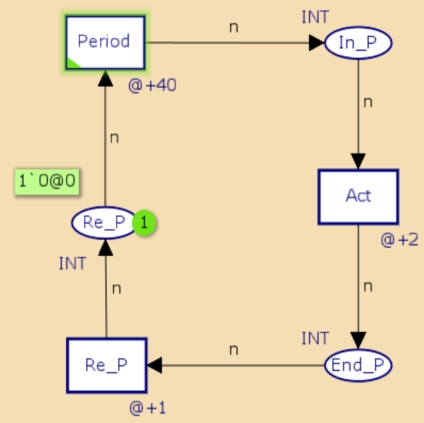
Representation in CPN of a periodic component.

**Figure 4.**
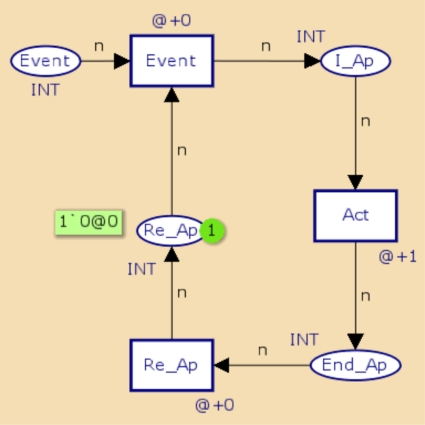
Representation in CPN of an aperiodic component.

Figures 3 and 4 show CPN representations of periodic and aperiodic components using a CPN Tool. These represent the behavior of periodic and aperiodic tasks described in C and Assembler languages, currently used for developing applications WSAN.

In the periodic object model, In P and End P represent the start and end states of the periodic activity, respectively, while the transition Act represents the development of the actions associated with the component. If necessary, more transitions can be placed to represent the development of the operations. The transition Re p and the place Re p indicate when the task is ready for a new implementation. Finally, the transition period indicates compliance of the implementation period of the component. The representation of the aperiodic component is very similar to the periodic component. Activation of the aperiodic component is performed by placing a mark at the site Event to generate the Event transition.

**Figure 5.**
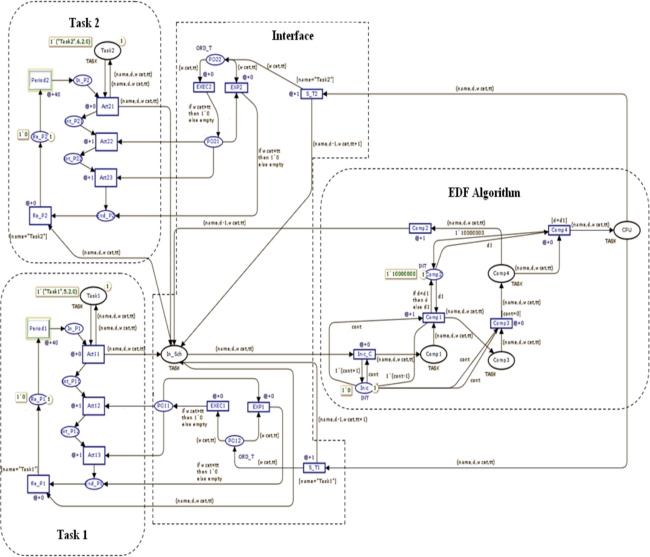
Representation in CPN of a node with two periodic tasks and an EDF scheduler.

Figure 5 shows the CPN model of a scheduler EDF that uses an interface to interact with each task. This process indicates if it won the competition for the processor (place CPU), or when it has completed its execution. In this case, the implementation of two periodic tasks is presented. The marks on the tasks have 4 fields (name, d, WCET, tt), representing the name of the task, deadline, worst-case execution time, and the time that it has been executed by the processor.

In the area of the EDF algorithm, the timer of each task is compared at each clock interval to schedule task’s in CPU, including those which may be running at the time. At Interface the task’s WCET is required for detecting blockages, it is possible by to verify if time in the CPU exceeds its WCET. The Table 1 shows the sequence of marks on the CPU during the simulation.

**Figure 6.**
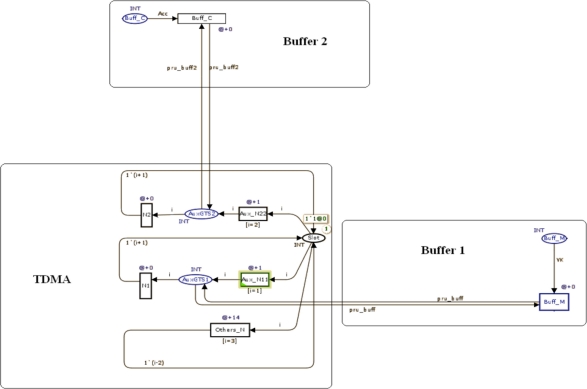
Representation in CPN of a TDMA network with two buffers.

Figure 6 shows the TDMA network model with 35 slots and two GTS slots, 1 and 2, which are assigned to nodes 1 and 2, respectively. This model represents the super-frame behavior of IEEE 802.15.4. Buff-M and BuffC represent the entry points to the network node’s buffers. Buff M and Buff C transitions are activated at the time instant nodes 1 or 2 have any data to transmit and the network slot scheduling matches its respective GTS. IN Act and IC represent the node’s input buffer to which the information is addressed.

## 5. Case Study

**Figure 7.**
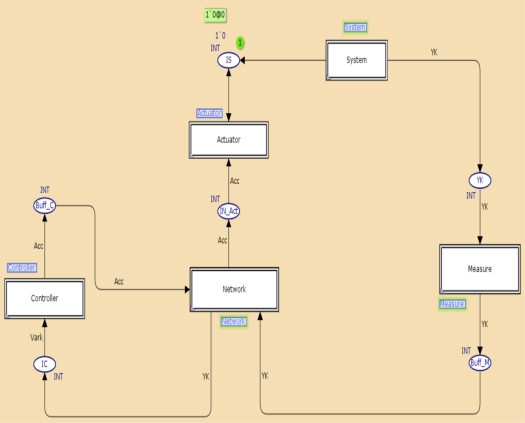
Hierarchical representation of the NCS.

In order to present the procedure proposed a NCS was analyzed. It was possible to represent the system to be controlled and the control algorithm at the same model. Figure 7 presents a hierarchical representation of the NCS.

### 5.1. General Considerations of the Study Case

We also consider that all frames are transmitted and received error-free, and that during the interval corresponding to a slot in the TDMA network, a node is capable of transmitting all data stored in the buffer. The latter assumption is made taking into account that the size of messages sent in industrial applications is small compared with the amount of data that can be sent in each message in current standard protocols (maximum payload in the physical layer PDU of 127 bytes for Zigbee and WirelessHART). The network configuration parameters for this case study are:

- Slot time duration in the TDMA network of 1ms.
- Slots Period of 35 ms.

### 5.2. NCS Considerations

About NCS the next assumptions were taken into account:

- Single input single output (SISO) system.
- The delay presented between the measurement and actuation is less than or equal to *T_S_*.
- The Measure task is activated periodically each *T_S_*. Controller and Actuator tasks are event driven.
- For the case study the system to be controlled was $G_{p(z)}=\frac{B}{Z-A}$, in which *y*(*k*) = *Ay*(*k* − 1) + *Bu*(*k* − 1).
- The following algorithm was used as a regulator $U_{\left(z\right)}=\frac{A_{\mathrm{CZ}}+B_{C}}{z-1}\varepsilon_{\left(z\right)}$, then *u*(*k*) = *u*(*k* − 1) + *Ace*(*k*) + *Bce*(*k* − 1)

**Figure 8.**
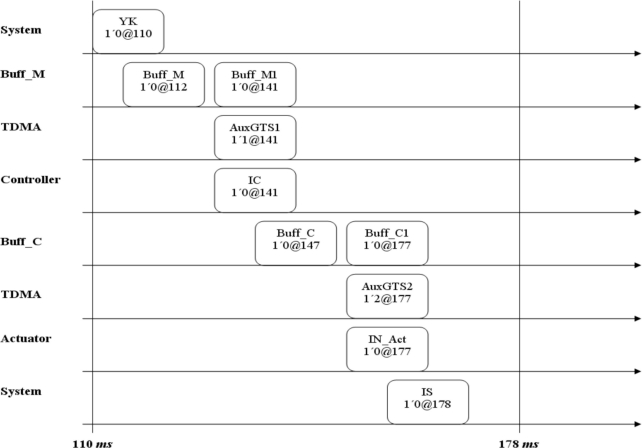
Simulations results from the CPN model between 110 and 178 ms.

The approach is as follows. The control application requires three tasks. The measure is represented by a periodic component, while the controller and actuator are represented by aperiodic components, which are executed on different nodes. The model presented in Figure 6 was used to represent the communications network, which is comprised of two GTS: GTS1 sends measures and GTS2 sends control actions. In this case, task schedulers were not considered because each node execute a single component. Figure 8 shows the sequence of events generated in the simulation of the case study. It can be seen that the delay generated from the moment when the simulation started until the moment the actuator acted was 68 ms, then deadline requirements were fulfilled.

**Figure 9.**
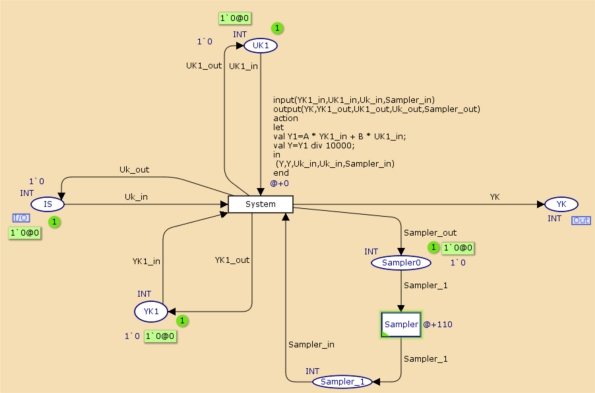
CPN model for the control system.

**Figure 10.**
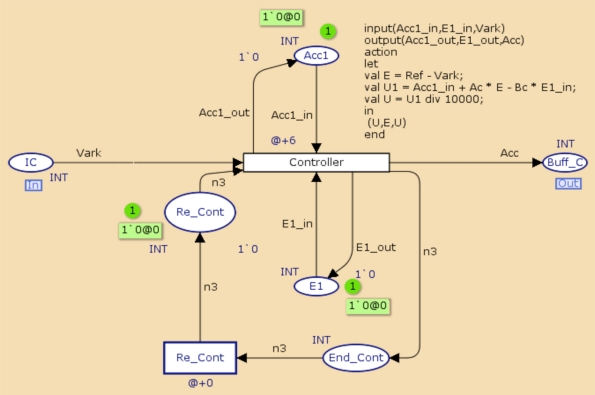
CPN model of the Controller task.

Figures 9 and 10 present the system model and the control task. In Figure 9, the System transition represents the system dynamics, which is sampled every 110 ms, and IS represents a zero-order hold for the input signal applied to the system. The controller transition in the controller task model represents the dynamics of the regulator.

### 5.3. Functional System Validation

The state-space results of the computer system (without the system model to control) using CPN Tools were:

- State Space. Nodes: 300, Arcs: 904, Status: Full. This indicates that the complete state space, composed of 300 nodes (states) and 904 arcs (occurrence of events), was generated.
- Best Integer Bounds: Upper = 1. This variable indicates that network places, including the communication buffer, will not have more than one mark in any system state.
- Home Markings: All. This indicates that any marking can be reached from any other marking. This assures the cyclical operation of the control system and that there is no blockage in the system.
- Dead Markings: None. This means that there are no markings where no transition is enabled, which is consistent with the previous results.
- Dead Transition Instances: None. This indicates that each transition has the potential to activate at least once.
- Live Transition Instances: All. This means that all transitions are reachable from any marking system, which again coincides with the cyclical behavior of the system. There are some other system properties that can be verified, but we only considered the most relevant ones related to the case study.

### 5.4. Temporal System Validation

The evaluating process technique used in this work is based on the decomposition of properties into simple terms each to be proven for earlier deadlines: once the validity of a simple term has been proven, the reachability space is cleared and the analysis is resumed from the reachable states that made the proof of the term possible. The reduction is given by the large amount of reachable states discard any time a term is proven: clearing the state space results in not building the successor states of all cleared reachable states. Terms are identified based on information on the problems easily accessible during verification. In this way, the reachability analysis technique can be used for analyzing much wider specifications. we establish a procedure to can suggest a possible decomposition of a certain property to be proven into two or more sub-properties. The proof of all of these sub-properties requires the exploration of a number of state much smaller than the direct proof of the initial property. The approach uses information from the designer for successive achievements in the exploration and reduce the number of state to be explored.

The criteria attempt to group situations with similar behavior and are based on general information provided by the design specifications and formal description of the model generated. The criteria helps to respond to the difficulties imposed by a models validation of WSAN using the architectural style developed, covering the functional and temporal aspects. Specifically, the following are the degrees of coverage:

- The simulation is started with a token in place YK at time 110.
- A token is located in place Buff_M at time 112 representing the delay in Measure node.
- Then, at time 141, the network allows to Measure node to send its information by using the GTS1.
- At time 147 the Controller node allocates a token in place Buff_C representing the delay as a consequence of calculate the control action.
- Then, at time 177, the network allows to Controller node to send its information by using the GTS2.
- Finally at time 178 the actuator actualize the control action in place IS.

Figure 10 represents the coverage criteria, which allows concluding that the system designed fulfilled with end-to-end time constrains proposed in the case study.

## 6. Conclusions

In this paper we present a minimal set of components for WSAN design and its representation in CPN, making it possible to verify behavioral properties and system structures. With the proposed components it was possible to completely validate a model and NCS. Future work will aim to increase the number of components proposed for the design, focusing mainly on implementation, thereby facilitating the design of these systems.

## Figures and Tables

**Table 1.** Marks sequence on the CPU between times instants 40 and 43.

Time instant	Marks
40	task1, 5, 2 ,0
41	task1, 4, 2, 1
42	task2, 4, 2, 0
43	task1, 3, 2, 1

**Table 2.** Marks sequence on the CPU between times instants 40 and 42.

Task	WCET(ms), low frequency
40	2
41	6
42	1
